# Thyroid Nodules: Emerging Trends in Detection and Visualization based on Citespace

**DOI:** 10.2174/1871530323666230822143549

**Published:** 2023-12-29

**Authors:** Wenyan Yao, Xiujuan Peng, Yunhui Guan, Xia Du, Conglong Xia, Feng Liu

**Affiliations:** 1 Shaanxi Institute of International Trade & Commerce, Xianyang, 712046, China;; 2 College of Pharmacy, Dali University, Dali, 671000, China;; 3 Shaanxi Academy of Traditional Chinese Medicine, Xi'an 710003, China;; 4 Shaanxi Buchang Pharmaceutical Co., Ltd., Xi'an, 710075, China

**Keywords:** Thyroid nodule, bibliometric analysis, WoSCC, malignant tumor, ultrasound, cytology, pathogenesis

## Abstract

**Background:**

Thyroid nodule (TN) is a highly prevalent clinical endocrine disease. Many countries have formed guidelines on the prevention and treatment of TN based on extensive research. However, there is a scarcity of TN-related literature based on bibliometrics.

**Objectives:**

This study aimed to evaluate the scientific achievements and progress of TN research from a global perspective by investigating the literature for 20 years through bibliometrics.

**Methods:**

We searched the literature on TN in the core collection of the Web of Science database from 2002 to 2021 and used the Citespace software to analyze the co-authorship, co-citation, and co-occurrence of countries, institutions, authors, keywords, and co-cited literature.

**Results:**

We retrieved 12319 documents related to TN. The literature on TN has been growing since 2002. The United States has contributed the largest proportion of TN papers (20.64%), followed by China, Italy, and South Korea. The United States ranked first in terms of centrality (0.38). Haugen BR, Gharib H, and Cibas ES are the top three most cited authors. The papers published in *Thyroid* were cited most frequently (7952 times). The most prominent keywords were management, cancer, fine needle aspiration, diagnosis, malignant tumor, thyroid cancer, ultrasound, biopsy, benign, surgery, ablation, and cytology. All keywords could be divided into three categories: diagnosis stratification, treatment, and cancer. As far as potential hot spots are concerned, the keywords that have recently burst strongly and are still continuing are: “Association Guideline” (2018-2021), “Radiofrequency Ablation” (2017-2021), “Classification” (2019-2021), and “Data System” (2017-2021).

**Conclusion:**

Based on the current trends, the number of publications on TN will continue to increase. The United States is the most active contributor to research in this field. Previous literature focused on stratification, cancer, surgery, and ablation, and there were different opinions on the stratification of diagnosis. There were relatively few studies on pathogenesis and treatment using medicine. More focus will be placed on association guidelines, radiofrequency ablation, classification, and data system, which may be the next popular topics in TN research.

## 1 INTRODUCTION

Thyroid nodule (TN), a common clinical thyroid disease, refers to abnormal growth of the parenchymatous thyroid cells, resulting in abnormal structure of one or more tissues in the thyroid gland. The most common types of TN are nodular goiter (non-tumorous), follicular adenoma (benign tumor), papillary carcinoma, and follicular carcinoma (mali- gnant tumor). TN is extremely common and can be detected in 65% of the population, of which only 5-15% may be cancerous [1]. Autopsy data from patients without a history of thyroid disease revealed a 50% prevalence of thyroid nodules [2].

TN genesis may be attributed to the amplification of thyroid heterogeneity due to genetic and/or epigenetic mechanisms [3]. Since iodine content is essential in thyroid hormone synthesis and secretion, iodine intake plays a key role in the pathogenesis of TN. Primarily, thyroid hyperplasia is the origin of TN, the development of which is related to age, gender, underlying inflammation, immune dysfunction, stress response, and radiation exposure. Nodular goiter may progress to the development of neoplasia, wherein somatic mutation may either lead to activation of oncogenes or growth arrest and colloid accumulation [3].

The literature regarding the diagnosis and treatment of TN is extensive, allowing us to determine a definite diagnosis and formulate a treatment plan accordingly. Kenneth *et al*. reviewed the process and essentials of the diagnosis and treatment of TN in detail [4] and reported that it is essential to stratify the risk of TN as early as possible for timely implementation of the most appropriate treatment measures and delay disease progression. Thereafter, several scholars have attempted to formulate a reasonable stratification method for thyroid nodules, in which ultrasound is the primary means of detection, risk stratification, and the guiding modality for subsequent biopsy and non-operative management [5]. The Thyroid Imaging Reporting and Data System (TI-RADS) developed by the American College of Radiology (ACR) has described distinct ultrasound features of TN, which are greatly helpful in differentiating between benign and malignant nodules. Additionally, mutations in certain proto-oncogenes, such as the BRAF, NRAS, KRAS, and HRAS, have also been reported to be associated with the development and progression of TN to distinguish benign TN from malignant ones [6].

Currently, the clinical treatment of TN mainly involves drugs, image-guided minimally invasive techniques (percutaneous absolute ethanol ablation, radiofrequency, laser, microwave ablation, and high-intensity focused ultrasound), and surgery. Drugs and minimally invasive techniques are often used in early disease, whereas surgery is generally opted for in malignant or suspected malignant nodules.

Over recent years, the thyroid nodule has received considerable attention and has undergone extensive research [7-8]. However, few attempts have been made to systematically investigate the scientific output and characterize the existing evidence from a global perspective. Therefore, it is necessary to adopt a suitable visualization method to reveal the global research status as well as future research trends and hotspots regarding TN. In this study, we performed a bibliometric analysis to systematically review studies on TN published between 2002 and 2021. Citespace, a data visualization software, was used to estimate the publication pattern of research on TN worldwide, assess the cooperation pattern between countries, institutions, and authors, and identify research trends and hotspots in this field.

## 2 MATERIALS AND METHODS

### 2.1 Source Database

For this study, we chose the Science Citation Index (SCI), an expanded, of the Web of Science Core Collection (WoSCC) database as the data source. The WoSCC database is one of the most comprehensive, systematic, and authoritative databases in the world and contains > 12,000 high-quality journals with global impact and is widely used in scientometric analysis and scientific literature visualization by a large number of studies [9, 10]. Accordingly, literature from the WoSCC was selected to scrutinize the present situation of TN research.

Citespace, designed by Chen [11], is a commonly used statistical analysis tool based on the Java environment. This citation visualization analysis software was gradually developed in the backdrop of scientometrics and data visualization, focusing on analyzing the potential knowledge contained in scientific literature. In Citespace, the structure, regularity, and distribution of scientific knowledge are presented through visualization to construct a map of the available scientific knowledge, which can then be used to explore the research hotspots, research fronts, knowledge base, main authors, and institutions of a certain research field. Simultaneously, it allows for predicting future development trends in a certain research field [12].

### 2.2 Retrieval Strategies

A systematic literature search was performed across the WoSCC for relevant publications using the search topic as TN and the language set as English. The timespan for data retrieval was set from 2002 to 2021. For manuscript types, only peer-reviewed original articles and reviews were included to ensure quality research; all other source types were excluded. Eventually, a total of 12319 publications on TN research were identified.

### 2.3 Data Collection

All results obtained from the WoSCC using the aforementioned search strategy were exported with full records, including titles, authors, abstracts, and cited references in .txt format. The literature search was performed on a single day, January 6^th^, 2022, to avoid the possibility of bias being introduced due to updates in the database. The data processed were imported into Citespace for systematic analysis.

Visual analysis of the data through Citespace V.5.8.R3 was performed based on the following three aspects: co-citation analysis of cited papers, analysis of scientific research cooperation network, and keyword co-occurrence network analysis. For all network visualizations, the following Citespace parameters were used: time slicing (2002-2021), years per slice (1 year), term source (title, abstract, author keywords, keywords plus), node types (choose one parameter at a time such as author, country, institution, reference, or keywords), selection criteria (top 50), and pruning (None). Further details of the software, utilization skills, and options are available in the Citespace manual.

**Scheme 1:**
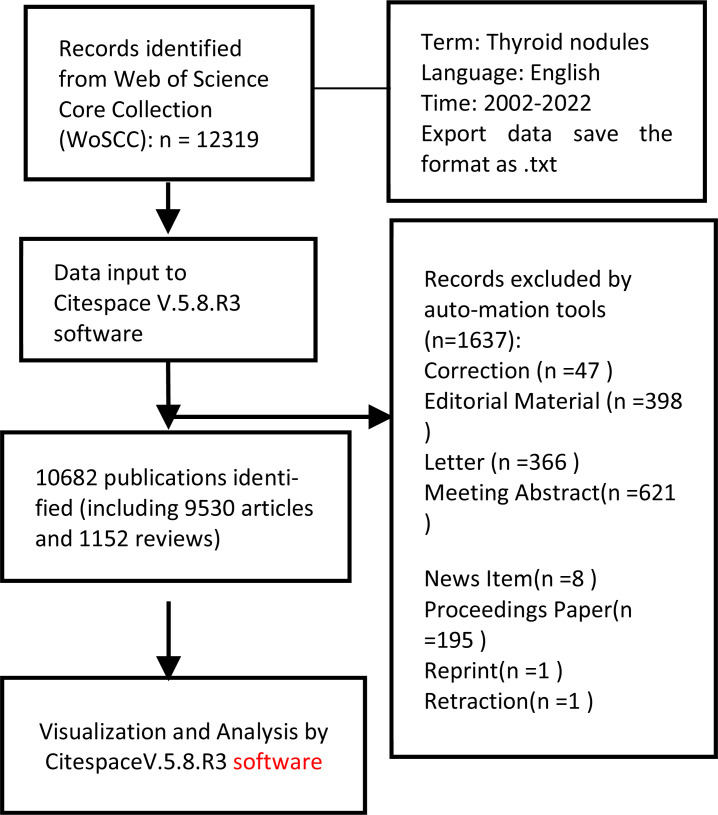
Flow chart for the selection of publications.

The flow chart for the selection of publications included in this study is as follows (Scheme **1**).

## 3 RESULTS

### 3.1 Publication Outputs and Citation Trends

We retrieved 12319 documents related to TN. As evident in Fig. (**1**), the number of related research articles and citations is proliferating which highlights the increasing attention being paid to different aspects of thyroid-related diseases.

### 3.2 Countries and institutional analysis

**Fig. (2):**
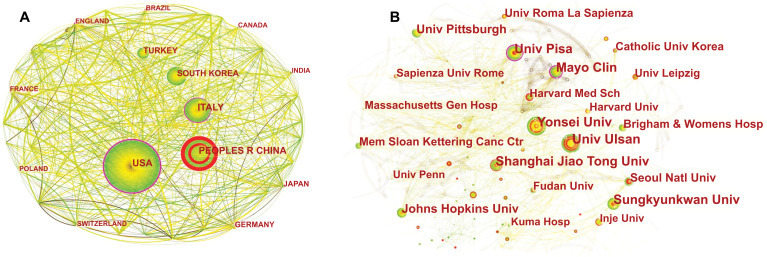
The collaboration network of countries (**A**) and institutions (**B**) on TN research from 2002 to 2021.

We analyzed each country's cooperation network map (Fig. **2A**) and used the Web of Science retrieval results to get the top 10 countries by the number of publications (Table **1**). The size of the node in Fig. (**2**) represents the number of published documents. The larger the node, the more the amount of published documents.

The centrality in Table **1** represents the importance of the node in the cooperative network. The higher the centrality, the more important the node is. We observed that the United States has published the most significant number of articles, followed by China, Italy, and South Korea, suggesting that these countries are leading the research in this field. The United States has high national centrality (which acts as a liaison in the partnership) and plays an intermediary role in the national cooperation network. On the other hand, centrality is relatively low in other countries. Table **1** also provides a list of the top 10 institutions leading the way in research related to TN. These institutions are primarily located in South Korea, Italy, China, and the United States, namely the University of Ulsan, Yonsei University, University of Pisa, Shanghai Jiao Tong University, Sungkyunkwan University, Mayo Clinic, University of Pittsburgh, Seoul National University, Johns Hopkins University, and Memorial Sloan Kettering Cancer Centre. This indicates that there is growing consideration toward TN across the globe, and these institutions were able to carry out more in-depth and lasting research with fruitful results owing to their dense network of cooperation and inter-institutional collaboration (Fig. **2B**).

### 3.3 Author and Keyword Analysis

Fig. (**3A**) presents the authors' co-occurrence graph, and the node size represents an author's publication volume and we can identify the authors with the biggest volumes of texts. The more numerous lines in the figure illustrate the closer cooperation between the authors. Table **2** contains a list of the top 10 authors by frequency, centrality, and burst value. Fig. (**3B**) contains the keyword co-occurrence graph; the most prominent keywords were management, carcinoma, fine needle aspiration, diagnosis, malignancy, thyroid cancer, ultrasound, biopsy, benign, surgery, and cytology. The keywords were categorized into three groups: diagnosis stratification, treatment, and cancer.

Table **3** presents the Top 20 keywords with the strongest citation bursts on TN research from 2002 to 2021. The blue line represents the time interval, while the red line shows the period in which a keyword had a burst. In all the studies, keywords with large mutation values that lasted until 2021 were association guideline, radiofrequency ablation, safety, management guideline, data system, classification, and guideline. These keywords may be suggestive of future trends in research on TN.

### 3.4 Co-citation Literature Analysis

**Fig. (4):**
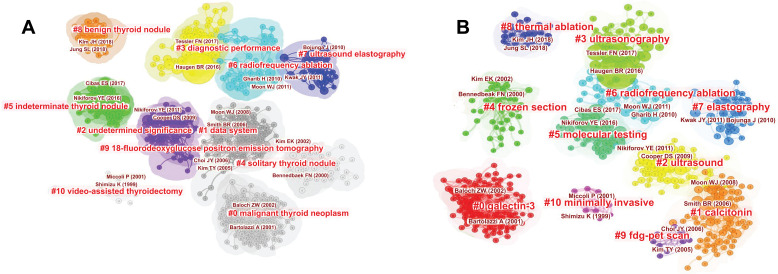
The co-citation network map of literatures by titles (**A**) and keywords (**B**) on TN research from 2002 to 2021.

A co-citation network diagram of the literature is presented in Figure **4**. The nodes in the graph represent the cited documents, which constitute the knowledge base of the research field, and the node size reflects the number of references. The more burst nodes a cluster contains, the more active the field, or an emerging trend of research. In Citespace, cluster labels are extracted from the nominal terms of citing documents, which can be considered a frontier area of research. Through these literature and cluster labels, readers can quickly understand the knowledge base and research frontier in this field. The tags extracted by the two classification methods of title and keyword are: (Fig. **4A**) malignant thyroid neoplasm, data system, undermined significance, diagnostic performance, solitary thyroid nodule, indeterminate thyroid nodule, radiofrequency ablation, ultrasound elastography, benign thyroid nodule, 18-fluorodeoxyglucose positron emission tomography (FDG-PET), and video-assisted thyroidectomy, (Fig. **4B**) galectin-3, calcitonin, ultrasound, ultrasonography, frozen section, molecular testing, radiofrequency ablation, elastography, thermal ablation, FDG-PET scan, and minimally invasive. Table **4** contains the ranking for citation frequency, centrality, and sigma value of highly cited papers; the larger the sigma value, the greater the influence of the literature.

### 3.5 Co-citation Journal Analysis

Fig. (**5**) presents a timeline view of journals. The clustering labels for the journals were ultrasound, ultrasonography, galectin-3, thyroidectomy, hyperthyroidism, and radio-guided surgery. The fields represented by these several labels all began in 2002, with ultrasound always being a hotspot. On analyzing the results of keywords, literature co-citations, and journal co-citations analyses, we found that the classification of TN by ultrasound and fine-needle aspiration has been a research hotspot to date. Thyroid was cited 7952 times with a centrality of 0.51, ranking first. Although the number of citations for the Journal of Nuclear Medicine journals was 366, the centrality was 0.15, ranking second. The burst detection results showed that the most cited journals in recent three years are the Journal of the American College of Radiology, European Thyroid Journal, and Scientific Reports-UK.

## 4 DISCUSSION

We observed that from 2002 to 2021, 558 institutions in 98 countries published articles related to thyroid nodules. The number of publications on TN research in the world continued to increase. In recent three years, the number of papers published each year exceeded 600. It can be inferred that research in this field is still a hot spot.

On analyzing the country-wise publications, the number of papers published in the United States in 2002 was 54, and it gradually increased to about 200 per year from 2018 to 2021. The U.S. is in the lead with a centrality of 0.38. Among the scientific research institutions in the United States, the centrality of the Mayo Clinic is 0.12, ranking first in the world. China is the country with the second most publications, but the centrality is only 0.01. The institution with the most published papers in China is Shanghai Jiao Tong University, with a centrality of 0.01. China also needs to invest more energy to increase its influence in this area.

As can be seen from Fig. (**3A**), the top three authors by the number of published papers are all from South Korea, namely JUNG HWAN BAEK of the University of Ulsan, JIN YOUNG KWAK of Yonsei University, and EUNKYUNG KIM of Konkuk University, and their main research areas are core needle biopsy. The burst detection results show that the top three authors with explosive growth in citations are: PIERPAOLO TRIMBOLI of the Università della Svizzeraitaliana, HUIXIONG XU of Tongji University, and Young Jun Choi of the University of Ulsan, whose main research fields are TIRADS, acoustic radiation force impulse (ARFI), and core needle biopsy. The fields of these six authors are all related to the diagnosis of thyroid nodules, which shows that diagnosis is still a research hotspot. The most cited author is Haugen BR from the University of Colorado, who was the first author of the 2015 American Thyroid Association (ATA) guidelines. The author with the highest centrality is Hossein Gharib from Mayo Clinic College of Medicine, who was the first author of guidelines for clinical practice for the diagnosis and management of TN-2016 update. It can be seen that the guideline plays an important role in the research in this field of TN.

Table **4** and Fig. (**4**) present the literature with the highest number of citations or values. Among the top ten cited kinds of literature in Table **4**, two works of literature proposed that excessive treatment should be prevented (Nikiforov YE, 2016; Alexander EK, 2012). With the development and application of various imaging technologies, such as ultrasound, the incidence rate of TN has increased explosively, which is related to diagnostic methods. The life of many TN patients and even cancer patients is not affected, so the problem of over-treatment deserves attention [13]. The remaining eight articles were on management guidelines and diagnostic data systems. It can be seen that management guidelines and diagnostic data systems are the focus of researchers.

In bibliometrics, the analysis of frequently occurring keywords can also reveal changing trends and major themes, Among them, the keywords with the strongest citation bursts can provide reasonable frontier predictions for TN research, which are crucial for understanding the development of the field. The keywords captured by Citespace are divided into the following three categories:

### 4.1 Diagnostic Stratification

In this research field, the prominent keywords captured by Citespace are diagnosis, fine needle aspiration, biopsy, ultrasonography, and classification. The detection methods of TN mainly include laboratory testing, ultrasonic testing, cytological testing, and molecular testing. Laboratory tests mainly include the detection of thyroid stimulating hormone (TSH) and thyroid hormone levels.

Sonography is the primary tool used for initial cancer risk stratification of TN. Many scholars around the world have conducted extensive and in-depth research in this field. Figure 4B shows that the works of literature on ultrasound are concentrated in three clusters (cluster2: ultrasound; cluster3: ultrasonography; cluster7: elastography). From 2010 to 2015, the burst keywords were “Differential diagnosis” and “US elastography” (Table **3**). US-elastography is a beneficial addition to the diagnosis of thyroid microcarcinoma by colour ultrasound. It can remarkably develop an accurate rate of the diagnosis of the disease. From 2015 to 2018, the burst keywords converted to “Shear wave elastography” (Table **3**). Shear Wave Elastography (SWE) is a useful imaging method that can be used with routine ultrasonography in the evaluation of thyroid in children [14]. A study showed that ARFI imaging is promising for malignant thyroid nodule prediction, with higher diagnostic performance than conventional US or EI. ARFI can be used to supplement conventional US to diagnose thyroid nodules in patients referred for surgery [15].

In terms of ultrasound report interpretation, the data system cluster has a larger number of documents (Fig. **4A**). From 2017 to 2021, “data system” became the burst keyword. The TI-RADS-based computer-aided (CAD) system performed well in the diagnosis of thyroid cancer. The CAD system can recognize 15 ultrasound features of thyroid nodules, most of which reached the level of 3 experienced radiologists [16]. In addition, a deep-learning AI model (ThyNet) to differentiate between malignant tumours and benign thyroid nodules aimed to investigate how ThyNet could help radiologists improve diagnostic performance and avoid unnecessary fine needle aspiration [17].

Whether to perform fine needle aspiration (FNA) depends on the ultrasonic results. From 2003 to 2013, “needle aspiration” was the burst keyword. FNA cytology is a common approach to evaluating thyroid nodules, although 20% to 30% of FNAs have indeterminate cytology, which hampers the appropriate management of these patients. From 2016-2019, the burst keyword was “Bethesda system”. Edmund S Cibas released the 2017 Bethesda Thyroid Cytopathology Reporting System. In the 2017 revision, the malignancy risks have been updated based on new (post-2010) data [18]. The latest study found that Midkine/free thyroxine (MK/FT4) and Midkine/thyroglobulin (MK/TG) in FNA washing fluid have diagnostic value for papillary thyroid carcinoma, especially thyroid nodules with uncertain cytology [19]. In addition, from 2006 to 2011, “Positron emission tomography (PET)” was the burst keyword. Cluster 9 in Fig. (**4B**) is a collection of literature related to 18-fluorodeoxy-glucose positron emission tomography (FDG-PET). In patients with thyroid nodules with indeterminate FNA, 18F-FDG PET/CT has a moderate ability to correctly discriminate malignant from benign lesions and could represent a reliable option to reduce unnecessary diagnostic surgeries. However, further studies using standardized criteria for interpretation are needed to confirm the reproducibility of these findings [20]. A study indicates that comprehensive genotyping of thyroid nodules using a broad next-generation sequencing (NGS) panel provides a highly accurate diagnosis for nodules with follicular (or oncocytic) neoplasm/susp-icious for a follicular (or oncocytic) neoplasm (FN/SFN) cytology and should facilitate the optimal management of these patients [21]. It is undeniable that fine-needle aspiration biopsy provides definite diagnostic information for evaluating thyroid nodules, but the cost of biopsy is relatively expensive, and it can also cause physical pain to patients. Therefore, novel diagnostic methods or auxiliary diagnostic methods are constantly being developed. When the strain ratio and elasticity score were used together for the differential diagnosis of thyroid nodules, more accurate results were obtained. Thus, combining both methods may be a promising alternative to fine needle aspiration biopsy in order to prevent unnecessary surgical interventions for suspected thyroid nodules [22].

Molecular detection focuses on the possible mutations of BRAF, RAS, TERT, TP53, and other related genes, and the existence of fusion genes [23]. Thyroid cancer is almost always present if a BRAF, TERT, or TP53 mutation is found on this test, or if a fusion gene is detected [24]. The label of cluster 5 in Fig. (**4B**) is “molecular test”. It can be seen that the number of documents in this cluster is relatively large. From 2005 to 2015, BRAF mutation became the burst keyword. Molecular analysis for a panel of mutations has significant diagnostic value for all categories of indeterminate cytology and can be helpful for more effective clinical management of these patients [25]. Another type of molecular test, gene expression analysis or gene expression classifier (GEC) uses proprietary algorithms to analyze the expression of specific genes in a gene panel and is designed to identify nodules that do not require surgery [1]. Usually, the diagnosis is based on ultrasonographic testing. If necessary, fine needle biopsy and molecular testing are carried out; however, the cost of molecular testing is relatively expensive. On the other hand, liquid biopsy, as a non-invasive diagnostic tool for body fluid genotyping, brings a new perspective on disease and therapy monitoring [26].

### 4.2 Treatment

From 2018 to 2021, “Association guideline” was the strongest burst keyword (Table **3**). So Association guideline is one of the recent hotspots. The United States issued the management guidelines for TN in 2015 and 2016 [13, 27], which provide comprehensive guidelines for TN from diagnosis to treatment, and propose future research directions. Other countries have also formulated guidelines in relevant fields, such as South Korea formulated the Radiofrequency Deviation Guideline [28], and Europe established the Ultrasound Malignancy Risk Strategy of Thyroid Nodules in Adults (The EU-TIRADS) [29]. Regarding the management of thyroid nodules, guidelines recommend follow-up for nodules without risk after diagnostic stratification; It is advised for those at risk to choose drug treatment (such as thyroid hormone) or image-guided minimally invasive techniques (percutaneous ethanol ablation, radiofrequency, laser, microwave ablation, and high-intensity focused ultrasound) as per the patient's condition. More serious cases need to consider surgery [27, 30].

The drug treatment for TN commonly involves the use of clinically available levothyroxine sodium. Low-dose levothyroxine is effective in the treatment of TN; however, higher doses may cause adverse reactions, such as palpitations, nausea, and vomiting, and long-term use may lead to osteoporosis and left ventricular enlargement [7]. Some authors suggest that thyroid hormone therapy is not recommended [1]. Some reports describe the use of metformin as a promising drug for treating thyroid diseases, but more studies are needed to evaluate the clinical significance of this drug in the treatment of TN [31]. On the other hand, Some studies have reported that natural extracts [32] and classic prescriptions of traditional Chinese medicine [33] have achieved good curative effects. An experimental study reported an 87.3% efficacy of the Xingqi Huatan Xiaoying decoction in the treatment of thyroid nodules, while the control group drug, levothyroxine sodium, was 67.3% efficacious [34]. Professor Mi Liehan used the Shugan Xiaoying decoction to treat nodular goiter with an efficacy of 94.6% [35]. Zhu *et al* used meta-analysis to compare the clinical effects of Integrated Chinese and Western Medicine on benign thyroid nodules and pointed out that integrated Chinese and Western medicine can be regarded as an alternative and effective treatment for benign thyroid nodules [36], indicating that the integration of Chinese and Western medicine has broad prospects.

Ablation is a highly effective and safe treatment for benign solid TN and may be considered a valid alternative to surgery [37-39]. Fig. (**4B**) shows that there are two clusters for ablation (cluster 6: radiofrequency ablation; cluster 8: thermal ablation). From 2017 to 2021, “Radiofrequency ablation was the burst keyword (Table **3**). So Radiofrequency ablation is one of the recent hotspots. A recent review demonstrated that both radiofrequency and laser ablation achieved significant volume reduction in the benign solid thyroid nodules, the efficacy of radiofrequency ablation was superior to that of laser ablation for volume reduction, and both intervention modalities were devoid of major complications [40]. Due to potential complications, thermal ablation procedures should be performed only by experienced operators. In addition, after a long-term follow-up of 3 years, the low power Microwave ablation (MWA) showed good safety and efficacy for the treatment of papillary thyroid microcarcinoma (PTMC). In addition to surgery and active surveillance, MWA might be another alternative for patients with PTMC [41].

Surgical treatment can eradicate TN, reduce the risk of cancer, and has an ideal curative effect. However, there is a risk of postoperative complications, such as bleeding, hypocalcemia, permanent hypothyroidism, recurrent laryngeal nerve, and parathyroid injury [1, 42]. The American Thyroid Association (ATA) guidelines also point out that most benign nodules do not support surgical treatment [13]. The main purpose of surgery or ablation is to remove the formed nodules; however, without correcting the cause of nodule formation, nodule recurrence is also possible. Therefore, more attention should be paid to the possible causes of thyroid nodules.

### 4.3 Cancer

Thyroid cancer is the most concerning issue for both patients and doctors when risk stratification is carried out. The treatment of thyroid cancer is usually surgical resection; however, it is aimed at determining the pathogenesis and carrying out targeted therapy. The study of the underlying mechanisms is conducive to risk stratification and targeted therapy [8]. In terms of cancer, the keywords captured by Citespace are cancer, malignancy, tumor, carcinoma, thyroid cancer, and papillary thyroid carcinoma. From 2002 to 2012, “Papillary carcinoma” and “Neoplasm” were the burst keywords (Table **3**). TC is a genetically simple disease with a relatively low somatic mutation burden in each tumor. Driver mutations are identified in more than 90% of TC [43]. The molecular pathogenesis of most TC cases involves an imbalance of the mitogen-activated protein kinase (MAPK) and phosphatidylinositol-3 kinase (PI3K) / Akt signaling pathway [44]. The United States and the European Union have approved four targeted therapy drugs: sorafenib, lenvatinib, vandetanib, and cabozantinib. A study summarized the recent clinical trials with targeted therapies that showed clinical benefits [45], and the study also pointed out that combination therapy of BRAFV600E inhibitor and anti-PD-1/PDL1 antibody is a promising therapeutic strategy for metastatic thyroid cancer.

Fig. (**4B**) shows that Galectin-3 and Calcitonin are the cluster labels of cluster 0 and cluster 1, respectively, both of which are closely related to the diagnosis and treatment of cancer. Galectin-3 (Gal-3), which has received significant attention for its utility as a diagnostic marker for thyroid cancer, represents the most well-studied molecular candidate for thyroid cancer diagnosis [46]. In 2019, Anti- Gal-3 antibodies are utilized as the targeting molecules of nanoparticles for the first time, which surprisingly increase intracellular DOX uptake by enhanced clathrin-mediated endocytosis, indicating that galectin-3 can be employed as a highly efficient target of drug delivery systems [47]. Calcitonin (Ct) is a sensitive diagnostic biomarker and one of the most important prognostic factors for Medullary Thyroid Cancer (MTC) outcomes. Patients who experienced postoperative Ct level normalization had a higher risk of disease recurrence than those with undetectable Ct levels after surgery [48]. Targeted therapies and immune therapies have demonstrated a significant clinical benefit. Further research is needed to provide strategies for better treatment of thyroid cancer.

Several limitations of our study should be pointed out. First, data on TN publications were only retrieved and collected from the WoSCC database, and publications in other databases may not have been studied. Second, although all searches were retrieved on January 6^th^, 2022, to avoid bias due to the daily update of the WoSCC database, the database remains in the open state as it is continuously receiving new studies.

## CONCLUSION

Based on current global trends, the number of publications on TN research will continue to increase. The United States is the most active contributor to research in this field. Previously, more attention was paid to diagnostic stratification, surgery, and ablation; however, there is no consensus on the most appropriate stratification method. More focus will be placed on association guideline, radiofrequency ablation, classification, and data system, which may be the next popular topics in TN research.

## Figures and Tables

**Fig. (1):**
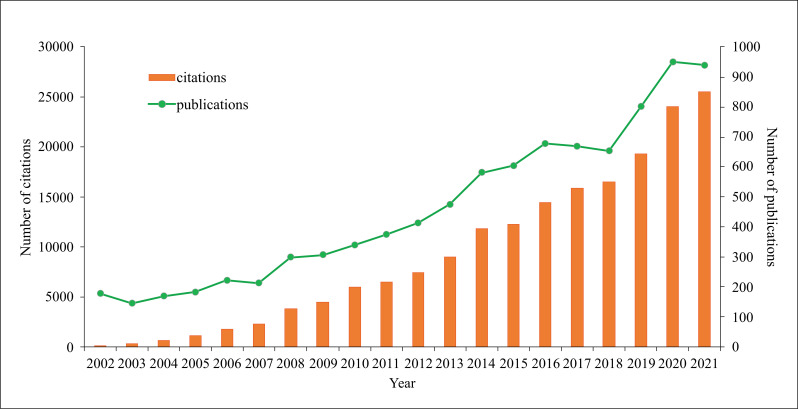
The number of articles published annually and the summed total citations of annual articles related to TN have been steadily increasing from 2002 to 2021.

**Fig. (3):**
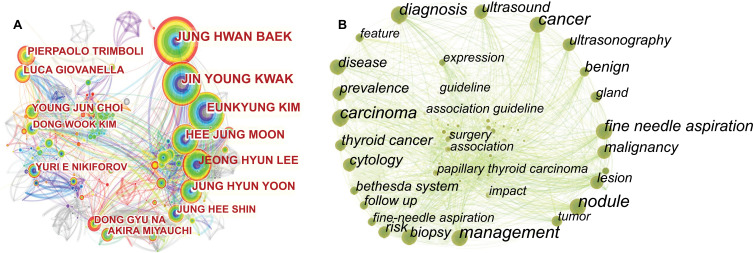
The network visualization map of authors (**A**) and keywords (**B**) on TN research from 2002 to 2021.

**Fig. (5):**
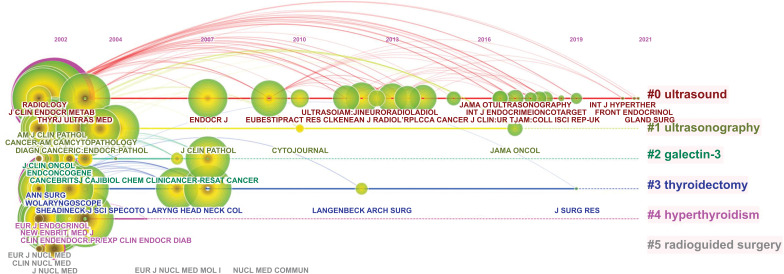
The timeline view of journals on TN research from 2002 to 2021.

**Table 1:** National and institutional ranking list on TN research from 2002 to 2021.

**Country Ranking**				**Institutional Ranking**			
**Countries**	**Publications**	**Centrality**	**Year**	**Institutions**	**Publications**	**Centrality**	**Year**
USA	2543	0.38	2002	University of Ulsan	251	0.03	2003
PEOPLES R CHINA	1682	0.01	2008	Yonsei University	235	0.04	2002
ITALY	1270	0.17	2002	University of Pisa	176	0.11	2002
SOUTH KOREA	1039	0.02	2007	Shanghai Jiao Tong University	168	0.01	2009
TURKEY	717	0.02	2007	Sungkyunkwan University	165	0.01	2003
GERMANY	426	0.06	2008	Mayo Clinic	149	0.12	2002
JAPAN	394	0.1	2007	University of Pittsburgh	134	0.03	2003
INDIA	310	0.02	2007	Seoul National University	128	0.01	2003
CANADA	289	0.02	2008	Johns Hopkins University	126	0.04	2003
BRAZIL	264	0.05	2007	Harvard University	104	0.06	2002

**Table 2:** Top 10 author by frequency, centrality, and burst value on TN research from 2002-2021.

**Author**	**Citation Counts**	**Author**	**Centrality**	**Author**	**Bursts**
Haugen BR	2376	Gharib H	86	Haugen BR	460.25
Gharib H	1988	Papini E	72	Cooper DS	201.95
Cibas ES	1635	Baloch ZW	60	Tessler FN	172.01
Cooper DS	1567	Alexander EK	52	Smith BR	128.00
Nikiforov YE	1169	Frates MC	48	Shin JH	101.52
Baloch ZW	1149	Mazzaferri EL	47	Grani G	88.08
Papini E	1065	Tan GH	46	Russ G	85.98
Hegedus L	1051	Hegedus L	44	Belfiore A	82.64
Mazzaferri EL	1014	Kim EK	41	Ha EJ	80.46
Frates MC	997	Nikiforova MN	41	Mazzaferri EL	80.33

**Table 3:** Top 20 Keywords with the Strongest Citation Bursts on TN research from 2002 to 2021.

**Keywords**	**Year**	**Strength**	**Begin**	**End**	**2002-2021**
Association guideline	2002	103.28	2018	2021	▂▂▂▂▂▂▂▂▂▂▂▂▂▂▂▂▃▃▃▃
Cytopathology	2002	59.92	2014	2018	▂▂▂▂▂▂▂▂▂▂▂▂▃▃▃▃▃▂▂▂
Papillary carcinoma	2002	58.66	2002	2012	▃▃▃▃▃▃▃▃▃▃▃▂▂▂▂▂▂▂▂▂
Radiofrequency ablation	2002	54.48	2017	2021	▂▂▂▂▂▂▂▂▂▂▂▂▂▂▂▃▃▃▃▃
Meta analysis	2002	48.8	2016	2018	▂▂▂▂▂▂▂▂▂▂▂▂▂▂▃▃▃▂▂▂
Undetermined significance	2002	46.07	2015	2018	▂▂▂▂▂▂▂▂▂▂▂▂▂▃▃▃▃▂▂▂
Gland	2002	44.42	2002	2009	▃▃▃▃▃▃▃▃▂▂▂▂▂▂▂▂▂▂▂▂
Safety	2002	43.01	2019	2021	▂▂▂▂▂▂▂▂▂▂▂▂▂▂▂▂▂▃▃▃
Graves disease	2002	42.29	2002	2013	▃▃▃▃▃▃▃▃▃▃▃▃▂▂▂▂▂▂▂▂
Management guideline	2002	42.28	2018	2021	▂▂▂▂▂▂▂▂▂▂▂▂▂▂▂▂▃▃▃▃
Data system	2002	42.01	2017	2021	▂▂▂▂▂▂▂▂▂▂▂▂▂▂▂▃▃▃▃▃
Classification	2002	41.18	2019	2021	▂▂▂▂▂▂▂▂▂▂▂▂▂▂▂▂▂▃▃▃
Positron emission tomography	2002	39.37	2006	2011	▂▂▂▂▃▃▃▃▃▃▂▂▂▂▂▂▂▂▂▂
Neoplasm	2002	37.96	2002	2012	▃▃▃▃▃▃▃▃▃▃▃▂▂▂▂▂▂▂▂▂
Us elastography	2002	37.42	2010	2014	▂▂▂▂▂▂▂▂▃▃▃▃▃▂▂▂▂▂▂▂
Shear wave elastography	2002	35.80	2015	2018	▂▂▂▂▂▂▂▂▂▂▂▂▂▃▃▃▃▂▂▂
BRAF mutation	2002	35.36	2005	2015	▂▂▂▃▃▃▃▃▃▃▃▃▃▃▂▂▂▂▂▂
Needle aspiration	2002	35.19	2003	2013	▂▃▃▃▃▃▃▃▃▃▃▃▂▂▂▂▂▂▂▂
Differential diagnosis	2002	34.80	2010	2015	▂▂▂▂▂▂▂▂▃▃▃▃▃▃▂▂▂▂▂▂
Guideline	2002	33.84	2019	2021	▂▂▂▂▂▂▂▂▂▂▂▂▂▂▂▂▂▃▃▃

**Table 4:** Top 10 highly cited articles on TN research from 2002 to 2021.

**Citation Counts**	**References**	**Centrality**	**References**	**Sigma**	**References**
2204	Haugen BR, 2016, THYROID, 26,1 DOI: 10.1089/thy.2015.0020. Cluster ID: 3	35	Nikiforov YE, 2011, J CLIN ENDOCR METAB, 96, 3390 DOI: 10.1210/jc.2011-1469. Cluster ID: 2	0.20	Alexander EK, 2003, ANN INTERN MED, 138, 315 DOI: 10.7326/0003-4819-138-4-200302180-00010. Cluster ID: 1
627	Cooper DS, 2009, THYROID, 19, 1167 DOI: 10.1089/thy.2009.0110. Cluster ID: 2	35	Alexander EK, 2003, ANN INTERN MED, 138, 315 DOI: 10.7326/0003-4819-138-4-200302180-00010. Cluster ID: 1	0.19	Baloch ZW, 2008, DIAGN CYTOPATHOL, 36, 425 DOI: 10.1002/dc.20830. Cluster ID: 2
503	Tessler FN, 2017, J AM COLL RADIOL, 14, 587 10.1016/j.jacr.2017.01.046. Cluster ID: 3	34	Alexander EK, 2012, NEW ENGL J MED, 367, 705 DOI: 10.1056/NEJMoa1203208. Cluster ID: 5	0.18	Nikiforov YE, 2011, J CLIN ENDOCR METAB, 96, 3390 DOI: 10.1210/jc.2011-1469. Cluster ID: 2
459	Gharib H, 2016, ENDOCR PRACT, 22,1 10.4158/EP161208.GL. Cluster ID: 3	34	Bartolazzi A, 2001, LANCET, 357, 1644 DOI: 10.1016/S0140-6736(00)04817-0. Cluster ID:3	0.16	Saggiorato E, 2005, ENDOCR-RELAT CANCER, 12, 305 DOI: 10.1677/erc.1.00944. Cluster ID: 0
394	Cibas ES, 2017, THYROID, 27, 1341 10.1089/thy.2017.0500. Cluster ID: 5	34	Baloch ZW, 2002, DIAGN CYTOPATHOL, 26, 41 DOI: 10.1002/dc.10043. Cluster ID: 0	0.15	Alexander EK, 2012, NEW ENGL J MED, 367, 705 DOI: 10.1056/NEJMoa1203208. Cluster ID: 5
367	Shin JH, 2016, KOREAN J RADIOL, 17, 370 10.3348/kjr.2016.17.3.370. Cluster ID: 3	34	Barden CB, 2003, CLIN CANCER RES, 9, 1792 https://aacrjournals.org/clincancerres/article/9/5/1792/204646/Classification-of-Follicular-Thyroid-Tumors-by. Cluster ID: 0	0.13	Haugen BR, 2016, THYROID, 26,1 DOI: 10.1089/thy.2015.0020. Cluster ID: 3
332	Nikiforov YE, 2016, JAMA ONCOL, 2, 1023 10.1001/jamaoncol.2016.0386. Cluster ID: 5	34	Kroll TG, 2000, SCIENCE, 289, 1357 DOI: 10.1126/science.289.5483.1357. Cluster ID: 0	0.13	Smith BR, 2006, THYROID, 16, 109 DOI: 10.1089/thy.2006.16.109. Cluster ID: 1
303	Russ G, 2017, EUR THYROID J, 6, 225 10.1159/000478927. Cluster ID: 3	33	Saggiorato E, 2001, J CLIN ENDOCR METAB, 86, 5152 DOI: 10.1210/jc.86.11.5152. Cluster ID: 0	0.13	Cooper DS, 2009, THYROID, 19, 1167 DOI: 10.1089/thy.2009.0110. Cluster ID: 2
298	Alexander EK, 2012, NEW ENGL J MED, 367, 705 10.1056/NEJMoa1203208. Cluster ID: 5	32	Baloch ZW, 2008, DIAGN CYTOPATHOL, 36, 425 DOI: 10.1002/dc.20830. Cluster ID: 2	0.13	Durante C, 2015, JAMA-J AM MED ASSOC, 313, 926 DOI: 10.1001/jama.2015.0956. Cluster ID: 3
277	Smith BR, 2006, THYROID, 16, 109 10.1089/thy.2006.16.109. Cluster ID: 1	32	Agrawal N, 2014, CELL, 159, 676 DOI: 10.1016/j.cell.2014.09.050. Cluster ID: 5	0.11	Nayar R, 2009, CANCER CYTOPATHOL, 117, 195 DOI: 10.1002/cncy.20029. Cluster ID: 2

## Data Availability

The dataset used and/or analyzed during the current study are available from the corresponding author, (XD), on reasonable request.

## LIST OF ABBREVIATIONS

ACR: American College of Radiology
ARFI: Acoustic Radiation Force Impulse
ATA: American Thyroid Association
CAD: Computer-Aided
Ct: Calcitonin
EI: Elasticity Imaging
EU-TIRADS: European Thyroid Imaging Reporting and Data System
FDG-PET: 18-Fluorodeoxyglucose Positron Emission tomography
FN: Follicular Neoplasm
GEC: Gene Expression Classifier
MAPK: Mitogen-Activated Protein Kinase
MK/FT4: Midkine/free thyroxine
MK/TG: Midkine/thyroglobulin
MTC: Medullary Thyroid Cancer
MWA: Microwave Ablation
PET: Positron Emission Tomography
PI3K: Phosphatidylinositol-3 Kinase
PTMC: Papillary Thyroid Microcarcinoma
SFN: Suspicious Follicular Neoplasm
SWE: Shear Wave Elastography
TI-RADS: Thyroid Imaging Reporting and Data System
TN: Thyroid Nodule
TSH: Thyroid-Stimulating Hormone
US: Ultrasound
WoSCC: Web of Science Core Collection
